# Standards, Processes, and Tools Used to Evaluate the Quality of Health Information Systems: Systematic Literature Review

**DOI:** 10.2196/26577

**Published:** 2022-03-08

**Authors:** René Noël, Carla Taramasco, Gastón Márquez

**Affiliations:** 1 Escuela de Ingeniería Informática, Universidad de Valparaíso Valparaíso Chile; 2 Departamento de Electrónica e Informática, Universidad Técnica Federico Santa María Concepción Chile

**Keywords:** health information systems, quality, standards, processes, metrics, systematic literature review

## Abstract

**Background:**

Evaluating health information system (HIS) quality is strategically advantageous for improving the quality of patient care. Nevertheless, few systematic studies have reported what methods, such as standards, processes, and tools, were proposed to evaluate HIS quality.

**Objective:**

This study aimed to identify and discuss the existing literature that describes standards, processes, and tools used to evaluate HIS quality.

**Methods:**

We conducted a systematic literature review using review guidelines focused on software and systems. We examined seven electronic databases—Scopus, ACM (Association for Computing Machinery), ScienceDirect, Google Scholar, IEEE Xplore, Web of Science, and PubMed—to search for and select primary studies.

**Results:**

Out of 782 papers, we identified 17 (2.2%) primary studies. We found that most of the primary studies addressed quality evaluation from a management perspective. On the other hand, there was little explicit and pragmatic evidence on the processes and tools that allowed for the evaluation of HIS quality.

**Conclusions:**

To promote quality evaluation of HISs, it is necessary to define mechanisms and methods that operationalize the standards in HISs. Additionally, it is necessary to create metrics that measure the quality of the most critical components and processes of HISs.

## Introduction

The quality of information systems represents the set of qualities and properties that characterize and determine the usefulness and existence of these systems [1] (eg, security, usability, scalability, and others). Quality can be interpreted as a set of characteristics that a product or service possesses, as well as its capacity to satisfy new and complex user requirements (eg, security in medical records [2]). This implies that the product or service complies with the specifications for which it has been designed and must conform to those as expressed by users and clients [3]. Some studies, such as Owens and Khazanchi [4], associate quality with (1) the explicitly stated functional and performance requirements, (2) the fully documented development standards, and (3) the implicit characteristics expected of any professionally developed system. On the other hand, the IEEE (Institute of Electrical and Electronics Engineers) defines quality as the degree to which a system, component, or process meets the specified requirements and the needs or expectations of the customer or user [5]. Both definitions denote that the emphasis of quality is on the specific requirements of the system and the pursuit of customer satisfaction.

Health information system (HIS) quality refers to whether a system’s internal and external specifications and the expectations of stakeholders are satisfied [6]. The development of informatics and technology has enabled health professionals to work with large volumes of data and information, as well as to transmit them smoothly. In turn, information from HISs can be used to drive decision-making, policy, research, and, ultimately, health outcomes [7]. In this regard, the use of health information technology improves the quality and effectiveness of health care. Additionally, it promotes individual and public health and increases diagnostic accuracy [8].

HIS quality can be measured in several forms, where the leading indicators are those related to patient care and the system’s components and structure [9]. Quality standards use methodologies for the design, programming, testing, and analysis of the developed system, with the objectives of offering (1) better reliability and maintainability that agree with the requirements demanded by users and (2) control of the quality of the system aiming at improving its effectiveness and efficiency [10]. In general, once the system has been validated as meeting the main functional requirements specified, the user will perform acceptance tests in order to deploy the system into the production environment.

HISs require methodologies and processes to evaluate their quality, since these systems map the diversity of health systems into explicit algorithmic functionalities, represented by software systems, which can inevitably produce problems in terms of efficiency or effectiveness in the work and daily activities of clinicians [11]. Traditionally, health services have been conceived as independent services where patients receive different types of medical care at different levels (ie, primary, secondary, and tertiary). This independence eventually leads to a lack of communication and coordination between services, which implies that the efficiency of an HIS is compromised [12]. Therefore, quality standards allow us to evaluate and standardize HISs in order to satisfy clinical requirements, define processes, reduce management problems, and develop HISs with high-quality standards. Although the range of quality standards in information systems, in general, is quite broad, there is little evidence of any compilation work that systematically identifies, evaluates, and describes evidence from primary studies related to quality standards in HISs. This situation makes it difficult to have a comprehensive perception of the most relevant aspects of the use and application of quality standards in HISs.

The importance and relevance of quality evaluation in HISs have been explored in several literature reviews. Villamor Ordozgoiti et al [13] conducted a literature review related to quality criteria in information and communication technologies in health care. The authors concluded that quality assessment that specifies health care systems’ requirements, including management, clinical, diagnostic, or monitoring, should be oriented toward the perspective of the institutions as users, clients, and acquirers of software. Sousa and Lopez [14] addressed the problem of usability regarding health care systems and how this problem can compromise the quality of the systems. For this reason, the authors conducted a systematic review regarding the usability of eHealth tools. The review results indicated that the poor usability of eHealth tools affects the possibility of adopting this type of system. Azad-Khaneghah et al [15] combined grey literature and academic literature reviews to evaluate mobile health (mHealth) apps’ usability and quality. The authors noted that most of the current mobile app quality rating scales have not been developed for the general public. Nouri et al [16] conducted a systematic review addressing the quality assessment of mHealth apps. As a result of the study, the authors mentioned the enormous heterogeneity in the evaluation criteria of mHealth apps in different studies. This may be due to the researchers’ various quality assessments or different definitions for each criterion. Triantafillou [17] conducted a narrative review on the quality management methods for electronic health records (EHRs). The results of this review indicated that there is substantial evidence that EHR systems contribute in various ways to improving quality management. Although there is a constant interest in quality assessment in several health care systems, to the best of our knowledge, few studies have explicitly addressed what standards, processes, and tools are used to assess HIS quality.

In this paper, we report the results of a systematic literature review (SLR) on methods used to evaluate the quality of HISs. The main objective of this review was to identify, characterize, and describe primary studies that complement the state-of-the-art of standards, processes, and tools used to evaluate HIS quality. We reviewed over 782 articles, from which we selected 17 (2.2%) primary studies. We analyzed, classified, and described each primary study in order to discuss the proposals for the quality evaluations of HISs. Based on our main objective, we defined the following research questions: What standards and certifications have been used to certify the quality of HISs? Which processes have been used to certify a software product? Which tools have been used by health software providers to certify a built software product?

The first research question addresses techniques and methods that allow us to describe patterns, models, or benchmarks to measure or evaluate quality of HISs. The second research question focuses mainly on describing phases or sets of activities that enable the evaluation of HIS quality. Finally, the third research question addresses technologies that facilitate the practical application of standards, certifications, and processes.

## Methods

### Identification

**Figure 1:**
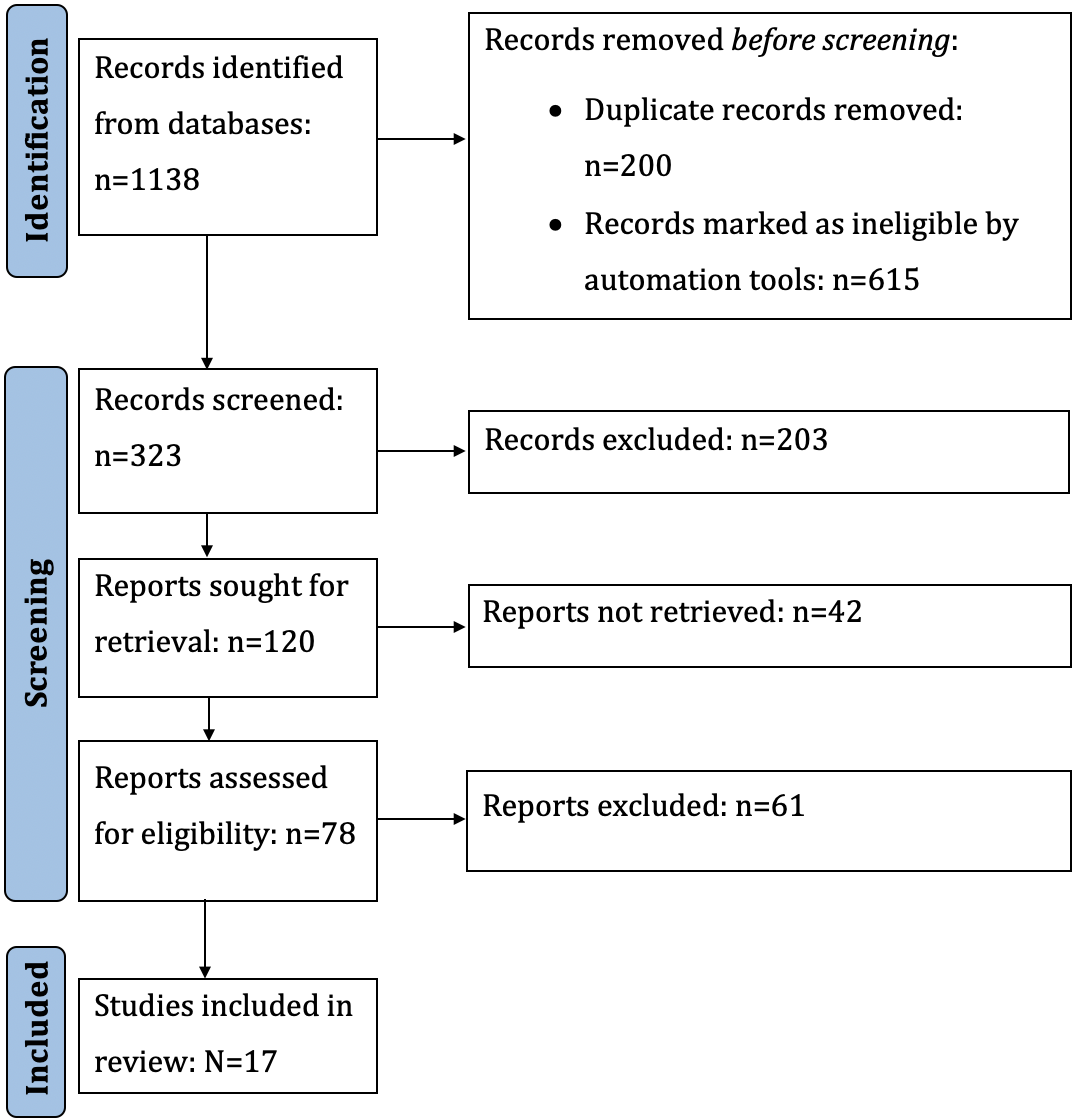
PRISMA flowchart of the selection of primary studies for the systematic literature review. PRISMA: Preferred Reporting Items for Systematic Reviews and Meta-Analyses.

We used the PRISMA (Preferred Reporting Items for Systematic Reviews and Meta-Analyses) statement [18] to conduct our SLR (Figure 1). Additionally, we used the PICO (population, intervention, comparison, and output) structure suggested by Petersen et al [19] to define a search string according to the population, intervention, comparison, and output. The following points describe the keywords for the PICO structure:

1. Population: articles related to HISs and synonyms (“health information system*” OR “e-health” OR “eHealth” OR “health software”).
2. Intervention: articles related to the quality assessment of HISs and synonyms (“certification” OR “testing” OR “validation” OR “verification” OR “assessment” OR “legalization” OR “quality”).
3. Comparison: there were no previous studies on the subject that could be used as a baseline for comparison since our objectives and goals were not the same.
4. Output: standards, tools, or processes applied in quality assessment (“process” OR “standard” OR “tool”).

We connected these concepts using “AND” and “OR” operators and obtained the following search string: (“health information system*” OR “e-health” OR “eHealth” OR “health software”) AND (“testing” OR “assessment” OR “validation” OR “verification” OR “certification”) AND (“process*” OR “standard*” OR “tool*”). We explored seven electronic databases—Scopus, ACM (Association for Computing Machinery), ScienceDirect, Google Scholar, IEEE Xplore, Web of Science, and PubMed—to search for and select primary studies.

### Screening

We screened primary studies using inclusion and exclusion criteria. We used the following inclusion criteria:

- The article is related to health
- The article provides HIS-related content and consolidated results from its research
- The article references or presents a standard, process, or tool
- The article addresses some quality attributes
- The article describes evaluation methods of HISs.

On the other hand, we applied the following exclusion criteria:

- The words “health” and “information system” are found, but they have no relation to our study
- The phrase “certification in health” is used, but its meaning is related to security
- The article is not related to standards, processes, or tools in the field of study
- The article is related to the quality attribute of interoperability.

### Quality Assessment

The purpose of the quality evaluation was to evaluate the importance of each selected document. Although the quality assessment did not affect the selection of primary studies [20], we describe the evaluation primarily to reflect the selected studies’ validity. According to each research question’s answer, we evaluated each paper with 2, 1, or 0 points. Then, we chose those papers that exceeded the 50% threshold. The studies selected through this evaluation will ensure that our conclusions from the extracted data have some support from adequate resources (see Multimedia Appendix 1 for more details on the quality assessment criteria).

## Results

### Overview

We identified 17 primary studies that were published in journals and in conference proceedings [21-37]. The scores of each selected primary study illustrate the quality and credibility of our study results. The average score was 74% (SD 0.11%), which means that the average quality of our study was acceptable (see Multimedia Appendix 2 for more details on the quality assessment results).

The study publication years ranged from 2004 to 2020. We did not find studies that were published in 2005, 2006, 2008, 2011, 2012, or 2014. We did not find primary studies published in workshop proceedings or book chapters (Multimedia Appendix 3).

Out of 17 primary studies, 59% (n=10) corresponded to research conducted in Europe. On the other hand, 24% (n=4), 12% (n=2), and 6% (n=1) of the studies corresponded to research conducted in Asia, the Americas, and Oceania, respectively (see Multimedia Appendix 4 for more detailed descriptions of the primary studies).

### Standards and Certifications

The primary studies described several types of standards and certifications that are used in HISs (Table 1).

**Table 1:** Standards and certifications used in the primary studies.

Study No.	Standards and certifications	Reference
1	Usability heuristics	[22]
2	Telemedicine quality control	[23]
3	IHE^a^ Connectathon, Q-REC^b^ and ProRec^c^, CCHIT^d^, and others	[24]
4	The IHE initiative	[25]
5	Lean and agile principles	[26]
6	ISO^e^/IEC^f^ 25010 standard	[27]
7	Custom certification framework	[28]
8	Custom usability principles applied in a case study	[29]
9	The uMARS^g^	[21]
10	The Constructive eHealth evaluation method	[30]
11	ISO 9241-210	[31]
12	The MDevSPICE framework	[32]
13	A care pathway data quality framework	[33]
14	The uMARS	[34]
15	The uMARS	[35]
16	The Medical Informatics Platform	[36]
17	The Medical Research Council framework	[37]

^a^IHE: Integrating the Healthcare Enterprise.

^b^Q-REC is a project entitled European Quality Labelling and Certification of Electronic Health Record Systems (EHRs).

^c^The ProRec initiative is a network of national nonprofit organizations (the “ProRec centres”) in Europe.

^d^CCHIT: Certification Commission for Health Information Technology.

^e^ISO: International Organization for Standardization.

^f^IEC: International Electrotechnical Commission.

^g^uMARS: end user version of the Mobile App Rating Scale.

A total of 65% (11/17) of the primary studies addressed standards that are related to quality management in HISs. Some of the primary studies, such as studies 1, 4, 5, and 10 (as numbered in Table 1), established that quality is based on the purposes and requirements established that must be met by any health care organization and the satisfaction of the needs of the people it serves. More precisely, HIS quality must allow for effective responses to health problems or situations that affect a population and its individuals, whether or not they identify them; HIS quality must also establish or apply the necessary diagnostic and therapeutic standards, procedures, and protocols to verify the medical instruments and means used. Studies 11 and 12 stated that translating quality policies to HISs can present diverse challenges that range from quality management to HIS implementation standards.

In addition, 35% (6/17) of the studies proposed metrics that allow for characterizing the standards. These metrics did not entirely represent a specific standard or certification, but rather they supported the evaluation of data control in telemedicine (study 2), the certification of EHRs (study 3), reliability in health-based mobile apps (studies 11, 15, and 17), and data management in medical platforms (study 16).

A total of 24% (4/17) of the primary studies addressed standards from a system perspective: 12% (2/17) addressed definition models, 6% (1/17) discussed design principles, and 6% (1/17) analyzed processes. In these studies, quality models and designs in HISs were discussed, but the implications of these proposals were not thoroughly discussed.

### Processes

Generally, the primary studies discussed little information about processes that allow for certifying the quality of software products. Some studies, such as study 17, indicated that some of the reasons why there are no documented or described cases of processes to certify software are the costs of the processes.

Study 3 mentioned the processes defined by the ISO 9126 (International Organization for Standardization) standard to evaluate the quality of EHRs. This ISO standard evaluates all the characteristics of a software product from internal and external perspectives. Following the same perspective of quality in software products, study 6 mentioned that several scientific studies described a considerable increase in the number of users who surf the internet to obtain health-related information online. For this reason, information-seeking behavior on the web results in a need to ensure that web-based portals meet basic quality standards. Therefore, study 6 described the experience of applying the ISO/IEC 25010 (International Electrotechnical Commission) quality assessment process to the e-Ebola Awareness System, an online health awareness portal. The process results provided some insights into the issues that negatively impacted the quality of the use of the portal, demanding attention and improvement.

A novel proposal, inspired by the ISO 9241-210 standard, was given by study 11. In this study, a human-centered design (HCD) approach was proposed to design connected health devices in order to ensure that user needs and requirements are considered throughout the design process. According to the authors, HCD is a multistage process that allows for several iterations of a design and subsequent updating of the requirements. Additionally, study 11 illustrated the implementation of an HCD by describing the techniques used to evaluate and develop usability and human factors in a case study addressing smartphone design and end user and stakeholder involvement.

### Tools

A total of 29% (5/17) of the primary studies used well-known tools to certify the quality of health software. Studies 6 and 11 used the System Usability Scale (SUS) instrument [38]. This scale provides a fast and reliable tool for measuring usability. It consists of a 10-item questionnaire with five answer options for respondents, ranging from “strongly agree” to “strongly disagree.”

In addition to using the SUS, study 11 also used the After-Scenario Questionnaire (ASQ) instrument [39]. This questionnaire uses three statements to assess a user’s perceived difficulty with a task in a usability test. Studies 1, 10, and 15 addressed the Nielsen heuristics [40], which are 10 guidelines that measure usability through human-computer interaction. These heuristics aim to create systems that are as user friendly as possible.

Additionally, studies 1, 9, and 10 used other tools that were specified concisely. Study 9 proposed a tool called the end user version of the Mobile App Rating Scale (uMARS), which consists of reliability testing of a version of the Mobile App Rating Scale (MARS) for end users [21].

## Discussion

### Overview

Concerning standards (research question 1) and processes (research question 2), quality management and metrics concentrated the largest number of primary studies. The studies addressed quality as part of HIS management, which implies little detail on how quality standards were addressed in HISs. Additionally, there was no evidence about using processes that help manage HIS quality standards. In addition, four primary studies (studies 2, 15, 16, and 17) addressed different perspectives of metrics and did not discuss, in depth, what types of processes they used to apply the metrics to HISs. Other primary studies, such as studies 7, 9, and 13, addressed standards related to processes, design principles, and the definition of models but did not discuss the processes that support these standards.

Regarding processes (research question 2) and tools (research question 3), it is also worth noting the little discussion of these topics in the primary studies. Unlike study 9, which addressed a custom tool, only studies 3, 6, and 11 explicitly described the tools they used to evaluate HIS quality and also included them in the processes (ie, ISO/IEC 25010 and ISO 9126). However, a significant number of primary studies did not fully address quality assessment tools and supporting processes.

Another aspect described by some primary studies, such as studies 9, 14, 15, and 17, was that many clinicians are now taking advantage of the potential of mobile apps to address specific health problems. This implies that there must be tools to assess the appropriateness of usability regarding mobile apps. Some of these tools point to user acceptability, ease of use, and identification of risks in the use of mobile apps among patients.

### Principal Findings

Our findings regarding HIS quality assessment revealed that there are several technical and social challenges to effectively achieving HIS quality objectives. More precisely, HIS quality assessment offers a method for evaluating the impact of changes in clinical processes that are embodied in systems. In general, the HIS represents an organized set of clinical functions involving people, data, activities, and overall material resources. These elements interact with each other to process data and information, including manual and automatic processes, in order to distribute them most appropriately within a given organization or entity based on its objectives.

Expanding the information on HIS quality requires the development of valid measurement instruments. The primary studies in this paper described some metrics, such as the SUS, Nielsen metrics, the ASQ, and others; however, these metrics only focus on one aspect of quality: usability. Usability in both HISs and the health sector is a critical attribute. Usability is defined as a measure of how well a specific user in a specific context can use a product or design to achieve a defined objective effectively, efficiently, and satisfactorily [40].

Other primary studies, such as studies 15 and 17, suggested that quality can be measured in how a user employs an HIS. In this regard, usability is one way to measure HIS quality. The primary studies mentioned several quality standards and processes, such as ISO 9126, ISO/IEC 62366:2015, and ISO/IEC 25010, that were used to evaluate HIS quality. These standards are composed of multidimensional attributes. For example, the ISO/IEC 25010 standard considers eight categories, as follows: (1) functional adequacy, (2) performance efficiency, (3) compatibility, (4) usability, (5) reliability, (6) security, (7) maintainability, and (8) portability. In turn, each category of the standard is divided into more quality attributes. Considering this example, it is natural to ask how HIS quality can be evaluated using the ISO/IEC 25010 standard as a reference. Extending this question to a more general scenario, another concern that emerges is what quality attributes are relevant in HISs.

The primary studies provided procedures and methods for evaluating HIS quality. Nevertheless, one aspect that we noticed is that there is no precise description of how to translate these standards into practice. Taking the example of the ISO/IEC 25010 standard, there is a considerable set of quality attributes that allow quality to be established in HISs. However, there is little information regarding success stories, case studies, or other empirical studies that describe the lessons learned about applying quality standards in HISs. This lack of information does not allow for the replication of results in other HISs in order to build a body of knowledge related to HIS quality. In addition, the primary studies described very discreetly what lessons they learned from applying quality standards to HISs. HISs involve not only technical aspects but also social aspects. The quality standards address the technical aspects of HIS quality but leave the social aspects of HIS quality to be addressed.

In the Results section, we described the metrics that were reported in the primary studies. However, these metrics addressed general aspects of information system usability. Although these metrics greatly contributed to evaluating HIS usability, they did not address other clinical aspects relevant to clinicians. Some metrics, such as NISTIR 7804 (National Institute of Standards and Technology Interagency/Internal Report) and Health-ITUES (Health Information Technology Usability Evaluation Scale), measure usability in specific HISs (eg, EHRs), but again, these studies fell short of measuring usability. Therefore, to expand the boundaries of quality evaluation, it is necessary to identify which quality attributes are most relevant to evaluating HIS quality. Once these attributes have been identified, it is possible to conduct research on defining precise metrics for evaluating HIS quality. Institutions such as the World Health Organization have proposed various tools to evaluate different aspects of HISs, such as organization, clinical staff, technologies, and others.

The findings identified in this review provide a first impression of the emerging challenges involved in HIS quality assessment. HISs are complex and, as such, considerable effort is required to evaluate the quality of a complex system. There is no doubt that more than one metric should be proposed to evaluate all HIS components, whether social or technical. However, it is also desirable to share the lessons learned regarding conducting HIS quality assessments. In this way, the results can be replicated to help health care institutions evaluate their HISs in order to improve the quality of care for their patients.

Additionally, our review revealed lines of research attempting to increase the body of knowledge on HIS quality assessments. Challenges related to identifying, describing, and characterizing relevant quality attributes for assessing HIS quality; creating and validating accurate quality metrics and instruments; and reporting success stories and empirical evidence regarding HIS quality assessments are just some possible research challenges that can be addressed by the community. Furthermore, the creation of a multidimensional metric to assess quality in HISs is also considered a challenge. Given that HISs are composed of several different components, proposing a metric that assesses quality in a cross-dimensional way requires further research.

The benefits of using processes to evaluate and certify HIS quality are positive. According to Love and Li [41], one of the main benefits is improving clinical process efficiency and effectiveness. This implies that the clinical services offered to patients are of good quality. From an internal management point of view, the processes help improve the internal communication capacity (ie, clinical services) and allow the different departments of a clinical institution to work together to satisfy patients’ needs and expectations. However, our study’s results revealed that the primary studies did not discuss, in depth, the use of processes to certify HISs. On this point, Love and Li [41] mentioned that applying quality certification processes can be highly demanding. For example, the costs of applying an HIS quality evaluation process are high, so such evaluations are often limited to organizations that have the resources for the evaluation. In addition, a large number of professionals are needed to conduct these assessments. This implies that the organization must have the resources to hire professionals and successfully conduct the assessment process. Under these scenarios, since health care institutions allocate their resources mainly to clinical and in-hospital management in order to care for patients, HIS evaluation does not necessarily rank high in managers’ priorities.

### Limitations

We critically reviewed the threats to the validity of our study. Because we conducted an SLR, this study suffers from the possible incompleteness of the search results and general publication bias. To analyze the threats to the validity of our study, we used the classification from Wohlin [42], which describes guidelines for classifying and mitigating threats to validity.

The threats to internal validity correspond to the factors that can affect the results of the study. In this regard, the bias in study selection is related to the potential bias in the search for articles in SLRs. To mitigate this threat, we used a robust literature review process on systems and software [20]. Additionally, we defined strict inclusion and exclusion criteria to select the primary studies. On the other hand, we are aware that the sample of studies obtained in this review was low (N=17). However, we performed several cross-check validations with three external collaborators to validate and evaluate each primary study. It is important to note that this SLR focused on HIS quality and not health quality. Therefore, after evaluating more than 700 studies, we determined that 17 primary studies satisfied our quality standards.

The threats to external validity are related to the restrictions that allow for the generalization of the results. The main threat is whether the primary studies represent HIS quality standards. To mitigate this threat, we invited health professionals from the Chilean National Center for Health Information Systems (CENS) to discuss and analyze each primary study in order to obtain feedback.

Regarding the threats to construction validity, these threats correspond to the generalization of the results to the concept underlying the execution of the SLR. The main threat is the subjectivity of our results. To mitigate this threat, we invited three external collaborators to support us in executing the SLR’s main steps and comparing the results independently.

### Conclusions

In this paper, we described the results of an SLR on HIS quality standards. We defined a rigorous process for identifying, characterizing, and evaluating this academic literature. As a result, we obtained 17 primary studies.

We identified five categories to classify standards. These categories are the definition of models, design principles, metrics, management, and processes. Most of the primary studies concentrated on the management category. We also realized that there is little information regarding processes that can be used to evaluate HIS quality. Some primary studies described evidence of ISO standards’ evaluation processes, such as ISO 9126 and ISO/IEC 25010, but most did not detail their information. Finally, the primary studies were not clear in explicitly describing what tools they used to certify HIS quality. The evidence for tools found in the primary studies suggests that these tools were usability evaluations.

Our findings point to primary studies agreeing that evaluating HIS quality is a relevant aspect of providing quality care to patients. However, several challenges compromise quality assessment. These challenges correspond to (1) the operationalization of quality certifications and standards in HISs, (2) the poor description of the metrics to measure HIS quality, and (3) the high demand for resources to conduct HIS quality assessments. To address these challenges, health care institutions must understand the importance of constantly assessing HIS quality. Defining standards, metrics, and processes for assessing quality provides countless benefits for HISs and contributes to creating quality-of-care models that ensure that patients receive appropriate treatments, thus minimizing the probability of errors.

## Appendix

Multimedia Appendix 1 Quality assessment criteria and the assignment of scores.

Multimedia Appendix 2 Quality assessment results.

Multimedia Appendix 3 Primary study distribution over the years. Numbers inside the circles represent the number of primary studies.

Multimedia Appendix 4 Descriptions of the primary studies.

## Abbreviations

ACM: Association for Computing Machinery
ASQ: After-Scenario Questionnaire
CENS: National Center for Health Information Systems
CORFO: Corporación de Fomento de la Producción
EHR: electronic health record
FONDECYT: Fondo Nacional de Desarrollo Científico y Tecnológico
FONDEF: Fondo de Fomento al Desarrollo Científico y Tecnológico
HCD: human-centered design
Health-ITUES: Health Information Technology Usability Evaluation Scale
HIS: health information system
IDeA: Investigación y Desarrollo en Acción
IEC: International Electrotechnical Commission
IEEE: Institute of Electrical and Electronics Engineers
ISO: International Organization for Standardization
MARS: Mobile App Rating Scale
mHealth: mobile health
NISTIR: National Institute of Standards and Technology Interagency/Internal Report
PICO: population, intervention, comparison, and output
PRISMA: Preferred Reporting Items for Systematic Reviews and Meta-Analyses
SLR: systematic literature review
SUS: System Usability Scale
uMARS: end user version of the Mobile App Rating Scale
