# Supplementary material for: Standards, Processes, and Tools Used to Evaluate the Quality of Health Information Systems: Systematic Literature Review
Source: J Med Internet Res. 2022 Mar 8;24(3):e26577. doi: 10.2196/26577 (PMC8941431; doi:10.2196/26577)
Supplement: Multimedia Appendix 1 [file jmir_v24i3e26577_app1.docx]

## Multimedia Appendix 1

Quality assessment criteria and the assignment of scores.

| ID | Quality assessment criterion | Answer | Points |
| --- | --- | --- | --- |
| QA1 | Quality Definition: Each work could have a different definition for Quality, but there are some international recognized definitions. | Standard | 2 |
|  |  | Framework or Technical Norm | 1 |
|  |  | Ad-hoc | 0 |
| QA2 | Method Description | Standards, well documented guidelines or software development methodologies. | 2 |
|  |  | Detailed ad-hoc method | 1 |
|  |  | Information not specified | 0 |
| QA3 | Rigor of the Method | Controlled Experiment | 2 |
|  |  | Study case, survey or quality assessment | 1 |
|  |  | Information not specified | 0 |
| QA4 | Adoption or Application | Applied in a real context | 2 |
|  |  | Applied in a simulated context | 1 |
|  |  | Information not specified | 0 |
| QA5 | Quantitative results | Results are explained from quantitative results obtained from the research | 2 |
|  |  | Results are explained from qualitative results obtained from the research | 1 |
|  |  | Information not specified | 0 |
| QA6 | Scope and limitations discussion | Describes threats that could invalidate the work | 2 |
|  |  | Describes scope and restrictions but does not specify threats | 1 |
|  |  | Information not specified | 0 |
