# Supplementary figures and images for: Standards, Processes, and Tools Used to Evaluate the Quality of Health Information Systems: Systematic Literature Review
Source: J Med Internet Res. 2022 Mar 8;24(3):e26577. doi: 10.2196/26577 (PMC8941431; doi:10.2196/26577)

## Multimedia Appendix 2

Quality assessment results.


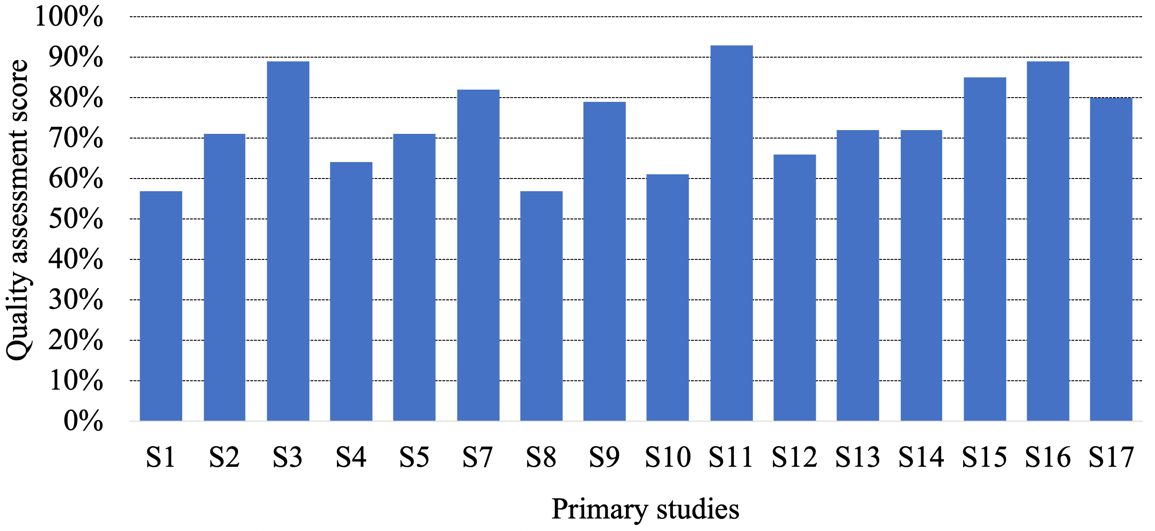

Supplement: Multimedia Appendix 2 [file jmir_v24i3e26577_app2.docx]
