# Supplementary material for: Standards, Processes, and Tools Used to Evaluate the Quality of Health Information Systems: Systematic Literature Review
Source: J Med Internet Res. 2022 Mar 8;24(3):e26577. doi: 10.2196/26577 (PMC8941431; doi:10.2196/26577)

## Multimedia Appendix 3

Primary study distribution over the years. Numbers inside circles represent the number of primary studies.


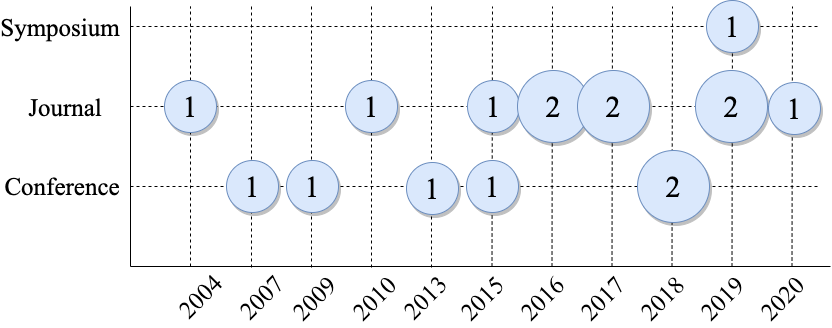

Supplement: Multimedia Appendix 3 [file jmir_v24i3e26577_app3.docx]
