# Supplementary material for: Standards, Processes, and Tools Used to Evaluate the Quality of Health Information Systems: Systematic Literature Review
Source: J Med Internet Res. 2022 Mar 8;24(3):e26577. doi: 10.2196/26577 (PMC8941431; doi:10.2196/26577)
Supplement: Multimedia Appendix 4 [file jmir_v24i3e26577_app4.docx]

## Multimedia Appendix 4

Primary studies description

| ID | Year | Authors | Country | Paper | Venue | Ref. |
| --- | --- | --- | --- | --- | --- | --- |
| S1 | 2004 | Kushniruk, Andre W., and Vimla L. Patel. | Canada | Cognitive and usability engineering methods for the evaluation of clinical information systems | Journal of biomedical informatics | [22] |
| S2 | 2007 | Giansanti, D., Morelli, S., & Macellari, V. | Italy | ﻿Experience at Italian National Institute of Health in the quality control in telemedicine: tools for gathering data information and quality assessing | International Conference of the IEEE Engineering in Medicine and Biology Society | [23] |
| S3 | 2009 | Hoerbst, A., Schabetsberger, T., Hackl, W., & Ammenwerth, E. | Austria | Requirements Regarding Quality Certification of Electronic Health Records | International Congress of the European Federation for Medical Informatics | [24] |
| S4 | 2010 | Virkanen, H., Mykkänen, J., & Kajaste, T. | Finland | ﻿Status of Interoperability Requirements related to IHE Integration Profiles in Finland | Finnish Journal of eHealth and eWelfare | [25] |
| S5 | 2013 | Manjunath, K. N., Jagadeesh, J., & Yogeesh, M. | India | Achieving quality product in a long term software product development in healthcare application using Lean and Agile principles | International Mutli-Conference on Automation, Computing, Communication, Control and Compressed Sensing (iMac4s) | [26] |
| S6 | 2015 | Hussain, A., & Mkpojiogu, E. O. | Malaysia | An Application of the ISO/IEC 25010 Standard in the Quality-in-Use Assessment of an Online Health Awareness System | Jurnal Teknologi | [27] |
| S7 | 2015 | Benedict, M., Burwitz, M., & Schlieter, H. | Germany | ﻿Certification of Service-oriented eHealth Platforms | International Joint Conference on Biomedical Engineering Systems and Technologies | [28] |
| S8 | 2016 | Unertl, K. M., Holden, R. J., & Lorenzi, N. M. | United States of America | Usability: Making It Real from Concepts to Implementation and End-User Adoption | Healthcare Information Management Systems | [29] |
| S9 | 2016 | Stoyanov, S. R., Hides, L., Kavanagh, D. J., & Wilson, H. | Australia | Development and Validation of the User Version of the Mobile Application Rating Scale (uMARS) | JMIR mHealth and uHealth | [21] |
| S10 | 2017 | Høstgaard, A. M. B., Bertelsen, P., & Nøhr, C. | Denmark | Constructive eHealth evaluation: lessons from evaluation of EHR development in 4 Danish hospitals | BMC medical informatics and decision making | [30] |
| S11 | 2017 | Harte, R., Quinlan, L. R., Glynn, L., Rodríguez-Molinero, A., Baker, P. M., Scharf, T., & ÓLaighin, G. | Ireland | Human-Centered Design Study: Enhancing the Usability of a Mobile Phone App in an Integrated Falls Risk Detection System for Use by Older Adult Users | JMIR mHealth and uHealth | [31] |
| S12 | 2018 | Özcan‐Top, Özden, and Fergal McCaffery. | Ireland | ﻿A Hybrid Assessment Approach for Medical Device Software Development Companies | Journal of Software: Evolution and Process | [32] |
| S13 | 2018 | Fox, F., Aggarwal, V. R., Whelton, H., & Johnson, O. | United Kingdom | ﻿A Data Quality Framework for Process Mining of Electronic Health Record Data | International Conference on Healthcare Informatics (ICHI) | [33] |
| S14 | 2019 | Li, Y., Ding, J., Wang, Y., Tang, C., & Zhang, P. | China | ﻿Nutrition-Related Mobile Apps in the China App Store: Assessment of Functionality and Quality | JMIR mHealth and uHealth | [34] |
| S15 | 2019 | Bardus, M., Ali, A., Demachkieh, F., & Hamadeh, G. | Lebanon | ﻿Assessing the Quality of Mobile Phone Apps for Weight Management: User-Centered Study With Employees From a Lebanese University | JMIR mHealth and uHealth | [35] |
| S16 | 2019 | Demiraj, A., Karozos, K., Spartalis, I., & Vassalos, V. | Greece | ﻿Meta-Data Management and Quality Control for the Medical Informatics Platform | International Database Applications & Engineering Symposium | [36] |
| S17 | 2020 | Hawley-Hague, H., Tacconi, C., Mellone, S., Martinez, E., Ford, C., Chiari, L., Helbostad, J. & Todd, C. | United Kingdom | ﻿Smartphone Apps to Support Falls Rehabilitation Exercise: App Development and Usability and Acceptability Study | JMIR mHealth and uHealth | [37] |
